# Interaction between Dipolar Lipid Headgroups and Charged Nanoparticles Mediated by Water Dipoles and Ions

**DOI:** 10.3390/ijms140815312

**Published:** 2013-07-24

**Authors:** Aljaž Velikonja, Poornima Budime Santhosh, Ekaterina Gongadze, Mukta Kulkarni, Kristina Eleršič, Šarka Perutkova, Veronika Kralj-Iglič, Nataša Poklar Ulrih, Aleš Iglič

**Affiliations:** 1 SMARTEH Research and Development of Electronic Controlling and Regulating Systems, Trg tigrovcev 1, Tolmin SI-5220, Slovenia; E-Mail: aljaz.velikonja@avelik.homeip.net; 2 Department of Food Science and Technology, Biotechnical Faculty, University of Ljubljana, Jamnikarjeva 101, Ljubljana SI-1000, Slovenia; E-Mails: poornima.budimesanthosh@bf.uni-lj.si (P.B.S.); natasa.poklar@bf.uni-lj.si (N.P.U.); 3 Laboratory of Biophysics, Faculty of Electrical Engineering, University of Ljubljana, Tržaška 25, Ljubljana SI-1000, Slovenia; E-Mails: ekaterina.gongadze@fe.uni-lj.si (E.G.); mukta.kulkarni@fe.uni-lj.si (M.K.); sarka.perutkova@fe.uni-lj.si (S.P.); 4 Jožef Stefan Institute, Jamova 39, Ljubljana SI-1000, Slovenia; E-Mail: kisstinca@hotmail.com; 5 Institute of Cell Biology, Faculty of Medicine, University of Ljubljana, Lipičeva 2, Ljubljana SI-1000, Slovenia; 6 Laboratory of Clinical Biophysics, Faculty of Health Studies, University of Ljubljana, Zdravstvena 5, Ljubljana SI-1000, Slovenia; E-Mail: veronika.kralj-iglic@fe.uni-lj.si; 7 Laboratory of Clinical Biophysics, Chair of Orthopaedic Surgery, Faculty of Medicine, University of Ljubljana, Zalǒska 9, Ljubljana SI-1000, Slovenia

**Keywords:** charged nanoparticles, lipids, osmotic pressure, dipolar zwitterionic headgroups, relative permittivity of water, orientational ordering

## Abstract

In this work, a theoretical model describing the interaction between a positively or negatively charged nanoparticle and neutral zwitterionic lipid bilayers is presented. It is shown that in the close vicinity of the positively charged nanoparticle, the zwitterionic lipid headgroups are less extended in the direction perpendicular to the membrane surface, while in the vicinity of the negatively charged nanoparticle, the headgroups are more extended. This result coincides with the calculated increase in the osmotic pressure between the zwitterionic lipid surface and positively charged nanoparticle and the decrease of osmotic pressure between the zwitterionic lipid surface and the negatively charged nanoparticle. Our theoretical predictions agree well with the experimentally determined fluidity of a lipid bilayer membrane in contact with positively or negatively charged nanoparticles. The prospective significance of the present work is mainly to contribute to better understanding of the interactions of charged nanoparticles with a zwitterionic lipid bilayer, which may be important in the efficient design of the lipid/nanoparticle nanostructures (like liposomes with encapsulated nanoparticles), which have diverse biomedical applications, including targeted therapy (drug delivery) and imaging of cancer cells.

## 1. Introduction

The basic building block of a cell membrane is a bilayer of lipid molecules with embedded carbohydrates and proteins [1–6]. The mechanical and electrical properties of a lipid bilayer [1,7–12] play an important role in many processes of the cell [1,13–17]. In experimental systems, the membrane lipid bilayer is usually in contact with an electrolyte solution composed of water molecules and positively and negatively charged salt ions [13,14]. Some of the lipids, like 1-palmitoyl-2-oleoyl-sn-glycero-3-phospho-L-serine (POPS), bears net negative electric charge, while the others, like glycerophospholipid 1,2-dipalmitoyl-sn-glycero-3-phosphocholine (DPPC), having dipolar (zwitterionic) headgroups, are electrically neutral [1–3,8]. The negatively charged lipid bilayer (headgroups) in contact with an electrolyte solution attracts cations and repels anions, and thus, an electric double layer is formed [18,19]. In the electric double layer, a strong variation of electric potential close to the negatively charged membrane was predicted [13,14,20–25]. In the case of a lipid bilayer composed of dipolar (zwitterionic) lipids, a substantial drop of the electric potential takes place within the headgroup region [13,26]. In the high electric field of dipolar lipid headgroups (see [13] and the references therein), the water dipoles are oriented towards the negatively charged plane (see, for example, [23,26–32]). Recently, it was shown that within a simple mean-field approach, due to the saturation effect in the orientational ordering of water dipoles, the relative permittivity in the zwitterionic headgroup region is decreased, while the corresponding electric potential becomes more negative [26].

**Figure 1:**
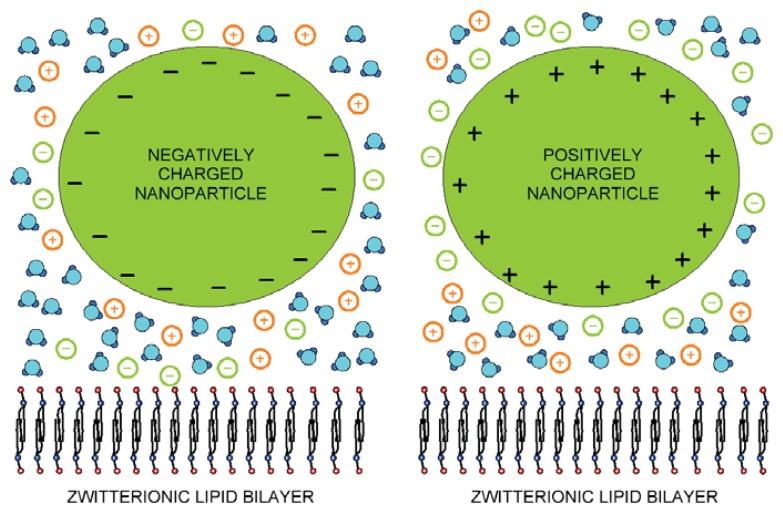
Schematic figure of dipolar zwitterionic lipid bilayer membrane in contact with the positively and negatively charged nanoparticles.

Liposomes encapsulated with the nanoparticles find enormous applications in various biomedical fields, such as cancer diagnosis, therapy and drug delivery [33]. As the usage of the nanoparticles is rapidly increasing, it becomes important to study the effect of differently charged nanoparticles on the cell membrane. The interaction of the nanoparticles with the lipid bilayer can alter the physical properties of the membrane, such as membrane fluidity, permeability and elasticity, and biological functions, such as cell signaling and transduction [34,35]. The biological and/or liposome membrane can be locally or globally deformed by the charged organic and inorganic nanoparticles attached to the membrane surface [36–39]. Among the charged organic nanoparticles, proteins are the biologically most important [38,40]. A number of proteins have been identified that directly bind and deform biological membranes [37,41–43]. The binding of proteins and other charged nanoparticles to the lipid bilayer of the cell membrane or membrane of liposomes is partially driven by electrostatic forces. Therefore, in this paper, the interaction between a negatively charged or dipolar flat lipid layer and positively or negatively charged nanoparticles (Figure 1) mediated by water dipoles and ions is studied within the mean-field approach using the modified Langevin-Poisson-Boltzmann (LPB) model [26,30]. An analytical expression for the osmotic pressure between the lipid headgroups and nanoparticles is derived, and the change of the average orientation lipid headgroups, due to the charged nanoparticle, is predicted. Through experimental study of the nanoparticle-induced changes in lipid bilayer fluidity, we intend to establish a correlation between the theoretical calculations and experimental results.

## 2. Interaction between Lipid Headgroups and Charged Nanoparticle

### 2.1. Space Dependence of Relative Permittivity within the Modified Langevin-Poisson-Boltzmann (MLPB) Model

Recently, the Langevin-Poisson-Boltzmann (LPB) model [30] was generalized to take into account the cavity field [31] in the saturation regime [26]. In this modified Langevin-Poisson-Boltzmann (MLPB) model [26], the electronic polarization of the water is taken into account by assuming that the point-like rigid (permanent) water dipole is embedded in the center of the sphere with a volume equal to the average volume of a water molecule in the electrolyte solution [31,44,45]. The permittivity of the single molecule’s water sphere is *n*^2^ = 1.33^2^, where *n* is the optical refractive index of water. The space dependency of permittivity within the MLPB model has the form [26,30]:

(1) $${ɛ}_{r}(x)=n^{2}+\frac{n_{0w}\;p_{0}}{{ɛ}_{0}}\;\left(\frac{2+n^{2}}{3}\right)\;\frac{L(\gamma p_{0}E(x)\beta )}{E(x)}$$

where *n*_0_*_w_* is the constant number density of water molecules, *p*_0_ is the magnitude of the water external dipole moment [31], *E*(*x*) is the magnitude of the electric field strength, *ɛ*_0_ is the permittivity of the free space, *β*= 1/*kT*, *kT* is the thermal energy and *ℒ*(*u*) = (coth(*u*) −1/*u*) is the Langevin function, while $\gamma =\frac{3}{2}\;\left(\frac{2+n^{2}}{3}\right)$ [31]. In the limit of *E*(*x*) →0, Equation (1) for *ɛ_r_*(*x*) gives the well-known Onsager expression: *ɛ_r,b_* ≅ *n*^2^ + (2 + *n*^2^/3)^2^*n*_0_*_w_p*_0_^2^*β*/2*ɛ*_0_. At room temperature (298K), the above equation predicts *ɛ_r_* = 78.5 for the bulk solution. The parameters, *p*_0_ and *n*_0_*_w/_N_A_*, are 3.1 Debye and 55 mol*/*L, respectively.

### 2.2. Osmotic Pressure between Two Planar Charged Surfaces

**Figure 2:**
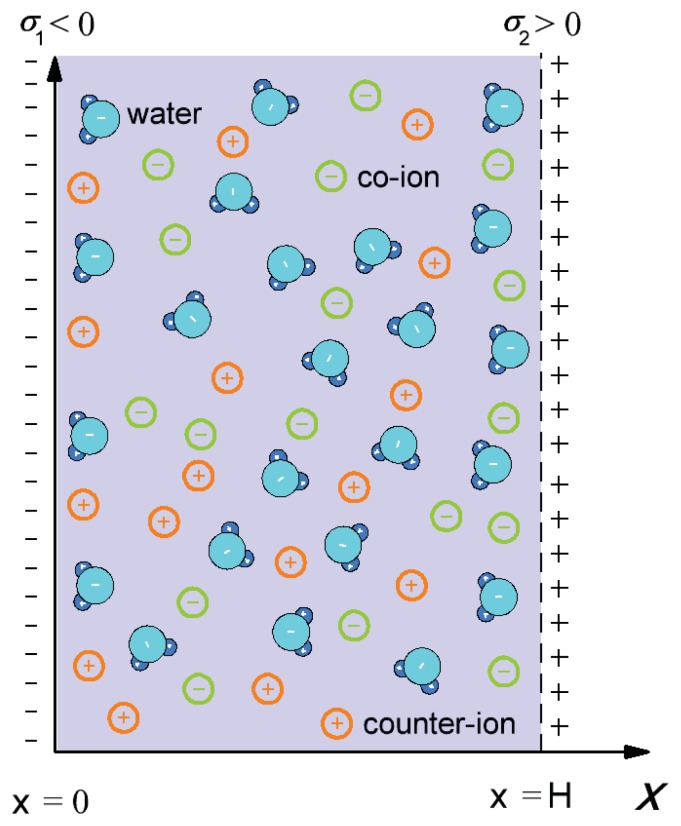
Schematic figure of the model of a negatively charged surface characterized by surface charge density, *σ*_1_, at *x* = 0 and a positively charged surface with surface charge density, *σ*_2_, at *x* = *H*.

In this subsection, we derive the expression for the osmotic pressure of electrolyte solution confined by two charged planar surfaces described in the model by surface charge density, *σ*_1_, at *x* = 0 and surface charge density, *σ*_2_, at *x* = *H* (see Figure 2). The space dependency of permittivity, *ɛ_r_*(*x*), is taken into account by Equation (1). The corresponding Poisson equation, *i.e.*, the MLPB equation, in a planar geometry can, thus, be written as: [26,30]:

(2) $$\frac{\text{d}}{\text{d}x}\;\left[{ɛ}_{0}\;{ɛ}_{r}(x)\frac{\text{d}\varphi }{\text{d}x}\right]=2\;e_{0}\;n_{0}\;\text{sinh }e_{0}\varphi \beta$$

where *ϕ*(*x*) is the electric potential, *e*_0_ is the unit charge, *n*_0_ is the bulk number density of salt anions and cations and *ɛ_r_*(*x*) is defined by Equation (1). The boundary conditions are (see, for example, [30]):

(3) $$\frac{\text{d}\varphi }{\text{d}x}(x=0)=-\frac{\sigma_{1}}{{ɛ}_{0}\;{ɛ}_{r}(x=0)}\;\;\;\frac{\text{d}\varphi }{\text{d}x}(x=H)=+\frac{\sigma_{2}}{{ɛ}_{0}\;{ɛ}_{r}(x=H)}$$

By integrating the MLPB Equation (2) and subtracting the corresponding bulk values from the local pressure between the lipid bilayer and nanoparticle surface, we obtain the expression for the osmotic pressure difference, Π= *P*_inner_ −*P*_bulk_ in the form (see Appendix):

(4) $$\begin{array}{c} \Pi =-\frac{1}{2}{ɛ}_{0}\;n^{2}\;E{\left(x\right)}^{2}+2\;n_{0}\;kT\;(\text{cosh}(-e_{0}\varphi (x)\beta )-1)-\\ -E(x)\;\left(\frac{2+n^{2}}{3}\right)\;n_{0w}\;p_{0}\;L(\gamma p_{0}E(x)\beta )+\left(\frac{2+n^{2}}{3}\right)\;\frac{n_{0w}}{\gamma \;\beta }\text{ln}\left(\frac{\text{sinh}(\gamma p_{0}E(x)\beta )}{\gamma p_{0}E(x)\beta }\right) \end{array}$$

Substituting the spatial number density distributions for cations and anions of electrolyte solution:

(5) $$n_{+}(x)=n_{0}\;\text{exp}(-e_{0}\varphi (x)\beta )\;,\;n_{-}(x)=n_{0}\;\text{exp}(e_{0}\varphi (x)\beta )$$

Equation (4) reads :

(6) $$\begin{array}{c} \Pi =-\frac{1}{2}{ɛ}_{0}\;n^{2}E{\left(x\right)}^{2}+kT\;(n_{-}(x)+n_{-}(x)-2\;n_{0})-\\ -E(x)\;\left(\frac{2+n^{2}}{3}\right)\;n_{0w}\;p_{0}\;L(\gamma p_{0}E(x)\beta )+\left(\frac{2+n^{2}}{3}\right)\;\frac{n_{0w}}{\gamma \;\beta }\text{ln}\left(\frac{\text{sinh}(\gamma p_{0}E(x)\beta )}{\gamma p_{0}E(x)\beta }\right) \end{array}$$

For small *γp*_0_*E*(*x*)*β*, we can expand the third and fourth term in Equation (6) into a Taylor series to get:

(7) $$\Pi =-\frac{1}{2}{ɛ}_{0}\;\left(n^{2}+{\left(\frac{2+n^{2}}{3}\right)}^{2}\;\frac{n_{0w}{p_{0}}^{2}\beta }{2\;{ɛ}_{0}}\right)\;E{\left(x\right)}^{2}+kT\;(n_{-}(x)+n_{-}(x)-2\;n_{0})$$

Using the Onsager expression for bulk relative permittivity, the above Equation (7) can be rewritten in the usual Poisson-Boltzmann (PB) form for osmotic pressure within the electric double layer theory [46]:

(8) $$\Pi =-\frac{1}{2}{ɛ}_{0}\;{ɛ}_{r,b}\;E{\left(x\right)}^{2}+kT\;(n_{-}(x)+n_{-}(x)-2\;n_{0})$$

In thermodynamic equilibrium, the value of the osmotic pressure is equal everywhere in the space between two charged surfaces (Figure 2); therefore we can calculate it at *x* = *H/*2 or at *x* = *H*. Using the boundary condition 3 Equation (8) becomes [46]:

(9) $$\Pi (x=H)=-\frac{\sigma_{2}^{2}}{2\;{ɛ}_{0}\;{ɛ}_{r,b}}+kT\;(n_{-}(x)+n_{-}(x)-2\;n_{0})$$

**Figure 3:**
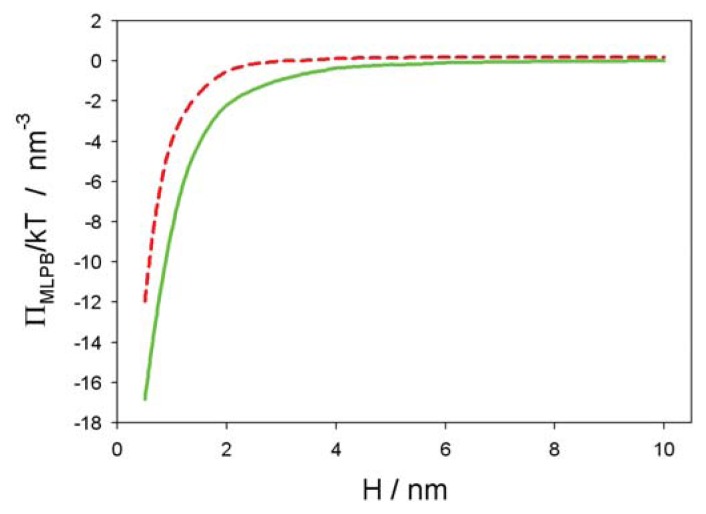
Osmotic pressure between between a negatively and positively surface (see Figure 2) as a function of the distance between both surfaces (*H*), calculated within the modified Langevin-Poisson-Boltzmann (MLPB) model for two values of the bulk salt concentration, *n*_0_*/N_A_* = 0.1 mol*/*L (dashed line) and *n*_0_*/N_A_* = 0.01 mol*/*L (full line). Other model parameters are : *σ*_1_ = −0.3 As*/*m^2^, *σ*_2_ = 0.3 As*/*m^2^, *T* = 298K, concentration of water, *n*_0_*_w_/N_A_* = 55 mol*/*L, and dipole moment of water, *p*_0_ = 3.1 Debye, where *N_A_* is the Avogadro number.

The MLPB equation (*i.e.*, Equation (2)) was solved numerically using MATLAB 2012a [26]) and COMSOL Multiphysics 4.3a [31]. Figure 3 shows the osmotic pressure between the negatively charged surface at *x* = 0 and the positively charged surface at *x* = *H* as a function of the decreasing distance between them (*H*). It can be seen that the decrease of Π(*H*) is more pronounced for the smaller values of the bulk concentration of salt. The predicted values of the osmotic pressure within the MLPB model differs from the corresponding values within the standard PB model, only at small distances, *H*. Within the MLPB model, the influence of the space variation of permittivity at both charged surfaces (see also [30,31]) on the osmotic pressure is not negligible.

## 3. Interaction between Dipolar Zwitterionic Lipid Headgroups and Charged Nanoparticle

**Figure 4:**
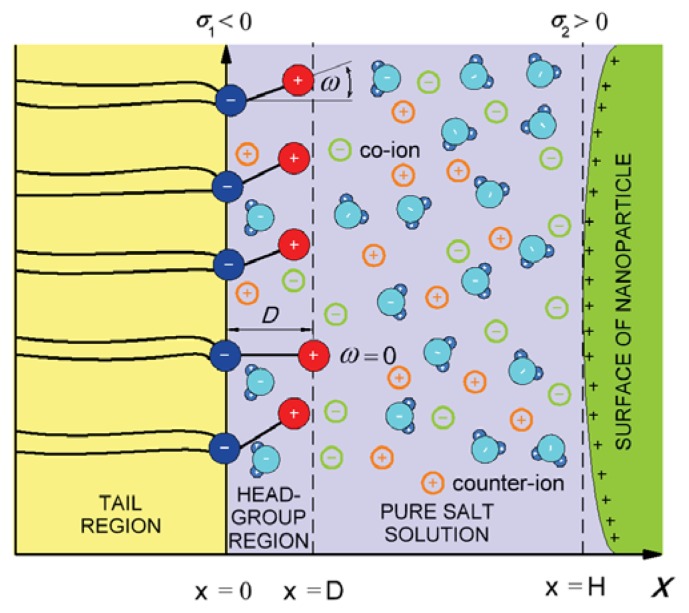
Negative charges of dipolar (zwitterionic) lipid headgroups are described by the surface charge density, *σ*_1_, at *x* = 0. The positive charges of the headgroups of dipolar lipids protrude in the electrolyte solution. Here, *D*, is the distance between the charges within the single dipolar lipid headgroup, and *ω* describes the orientation angle of the dipole within the single headgroup. The positive charge of the interacting nanoparticle is described by the surface charge density, *σ*_2_.

In the model, the zwitterionic dipolar lipid headgroup composed of a positively charged trimethylammonium group and a negatively charged carboxyl group (at neutral pH) is described by two charges at fixed distance, *D* (Figure 4) [26]. The negative charges of the phosphate groups of dipolar (zwitterionic) lipids are described by negative surface charge density, *σ*_1_ at *x* = 0, while the positive surface charge of the nanoparticle (Figure 1) is approximated by the planar charged surface at *x* = *H* with the surface charged density, *σ*_2_. The corresponding Poisson equation in a planar geometry can be then written in the form [26]:

(10) $$\frac{\text{d}}{\text{d}x}\;\left[{ɛ}_{0}\;{ɛ}_{r}(x)\frac{\text{d}\varphi }{\text{d}x}\right]=2\;e_{0}\;n_{0}\;\text{sinh }e_{0}\varphi \beta -\rho_{Zw}(x)$$

where *ρ_Zw_*(*x*) is the macroscopic (net) volume charge density of positive charges of dipolar (zwitterionic) headgroups [26]:

(11) $$\rho_{Zw}(0<x\leq D)=\frac{|\sigma_{1}|P(x)}{D}\;\;\;\text{and }\;\;\rho_{Zw}(x>D)=0$$

where *℘*(*x*) is the probability density function [26]:

(12) $$P(x)=\Lambda \frac{\alpha \;\text{exp}(-e_{0}\varphi (x)\beta )}{\alpha \;\text{exp}(-e_{0}\varphi (x)\beta )+1}$$

where Λ is determined from normalization condition, $\frac{1}{D}\int_{0}^{D}P(x)\;\text{d}x=1$. The corresponding boundary conditions, as described in [25,26], should be taken into account.

**Figure 5:**
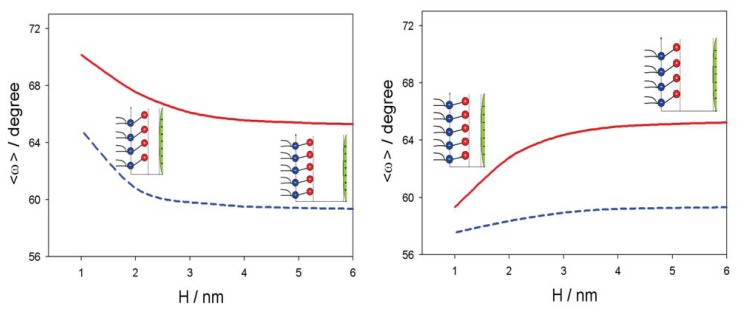
Average lipid dipolar headgroup orientation angle, *< ω >* (see, also, Figure 4), as a function of the distance (*H*) between the plane of the phosphate groups of dipolar (zwitterionic) headgroups and the surface of positively (**left** panel) and negatively (**right** panel) charged nanoparticle for the bulk concentration of salt, *n*_0_*/N_A_* = 0.1 mol/L, and two values of parameter, *α*: 0.5 (full line) and five (dashed line). The values of the model parameters are: the dipole moment of water, *p*_0_ = 3.1 Debye, and concentration of water, *n*_0_*_w/_N_A_* = 55 mol/L.

**Figure 6:**
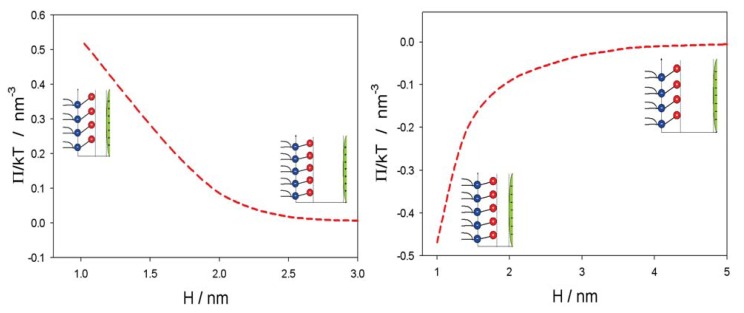
Osmotic pressure between the plane of the phosphate groups of dipolar (zwitterionic) headgroups and the surface of positively (**left** panel) and negatively (**right** panel) charged nanoparticle, as a function of the distance (*H*) (see, also, Figures 4 and 5) calculated for *α* = 5 and the bulk concentration of salt, *n*_0/_*N_A_* = 0.01 mol/L, by using Equation (9). The values of other model parameters are the same as in Figure 5.

Figure 5 shows the influence of approaching positively and negatively charged nanoparticles on the average orientation of the lipid dipolar headgroup angle (*< ω >*). As expected, the value of *< ω >* increases with decreasing *H*, due to electrostatic repulsion between the positive charged parts of the lipid headgroups and the positively charged nanoparticles. In accordance, also, the osmotic pressure between the headgroups and nanoparticle is increased with decreased *H* in the case of a positively charged nanoparticle and decreased in the case of a negatively charged nanoparticle, as presented in Figure 6.

## 4. Experimental Results

Membrane fluidity denotes the viscosity of the phospholipid bilayer of a cell, and fluidity enables the free mobility of the lipids and protein molecules in a cell membrane [47]. Alteration in the membrane fluidity can affect various membrane associated functions of the cell. Fluidity of a cell membrane is affected by various factors, such as temperature, osmotic pressure, length of membrane fatty acid chains, cholesterol, nanoparticles and the degree of saturation of the lipids in the membrane [48]. In this work, small unilamellar vesicles were prepared to measure the bilayer fluidity in the presence of positively and negatively charged nanoparticles (NPs). The fluidity of the lipid bilayer membrane of small unilamellar vesicles was determined by measuring the fluorescence anisotropy, which is directly proportional to the lipid ordering in the membrane and inversely proportional to the membrane fluidity. As the membrane becomes more fluid, the mobility of the fluorescent dye (DPH) incorporated into the bilayer also increases, whereas the intensity of the fluorescence emission from the dye decreases. Hence, increased anisotropy values indicate that the membrane fluidity is decreased, and the lipids are in a more ordered (liquid) phase. On the other hand, decreased anisotropy values denote increased membrane fluidity, and the bilayer lipids are in a less ordered (liquid) phase.

### 4.1. Synthesis of Nanoparticles

**Figure 7:**
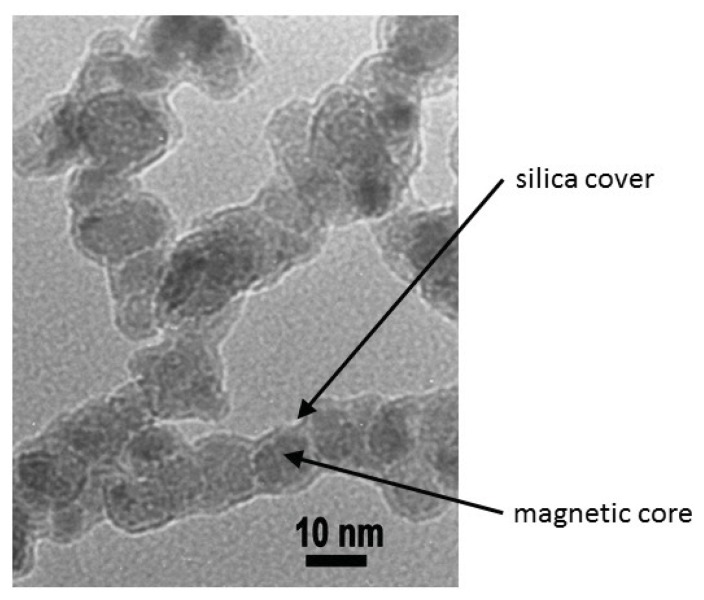
TEM image of superparamagnetic maghemite nanoparticles (*γ* −Fe_2_O_3_), covered with 20 nm thick silica.

Superparamagnetic maghemite nanoparticles (*γ* − Fe_2_O_3_) were synthesized through a controlled chemical co-precipitation method. An aqueous solution of iron (II) sulfate heptahydrate (FeSO_4_ ·7H_2_O) and iron (III) sulphate hydrate (Fe_2_(SO_4_)_3_ · H_2_O) was prepared at acidic conditions (purchased from Alfa Aesar). The co-precipitation method has been used as a two step process. In the first step, iron hydroxides were precipitated in an alkaline medium during the reaction between the aqueous solution of metal salts and an aqueous solution of ammonium hydroxide. The corresponding metal hydroxides were precipitated during the reaction between the alkaline precipitating reagent and the mixture of metal salts and, subsequently, oxidized in air to form *γ* − Fe_2_O_3_ in the second step of the process. The temperature for this process was set constant at 25 ºC for 1 h. After the reaction, nanoparticles were washed with diluted ammonia solution at pH 10 several times and 5 mg/mL of a solution of citric acid (purchased from Sigma-Aldrich) during stirring to prepare stable aqueous suspension. Particles were additionally coated with SiO_2_ cover and functionalized with different groups. In order to stabilize the aqueous suspension of the magnetic nanoparticles, the particles were coated with a silica layer prepared by hydrolysis and polycondensation of tetraethyl orthosilicate (TEOS, purchased from Alfa Aesar) using alkaline medium. TEOS was added to the mixture by dropwise addition for 1 h and, after that, rigorously stirred for 3 h at room temperature. Using silica cover helps to prevent agglomeration, as well as provides an easily modifiable surface for creating different charges or groups on the surface of the nanoparticles. As shown in recent publications the cover is also biocompatible regarding cell viability studies [49]. Additional amino [ ${\text{NH}}_{3}^{+}$] groups were added to their surface to create a positive charge using grafting with 3-(2-aminoethylamino) propylmethyldimethoxysilane (APMS, 97 %), purchased from Alfa Aesar. The similarly charged particles reduce the rate of aggregation, due to strong electrostatic repulsions, thereby ensuring increased stability. The nanoparticles were characterized for size and morphology using Transmission Electron Microscopy (TEM) model JEM 2100 at 200 kV from JEOL. The size of the synthesized *γ* −Fe_2_O_3_ nanoparticles was found to be 10±2 nm, observed by TEM analysis, as shown in Figure 7.

The negatively charged cobalt ferrite nanoparticles were synthesized by co-precipitating the stoichiometric mixtures of Fe(NO_3_)_6_ ·9H_2_O and Co(NO_3_)_2_ ·6H_2_O in aqueous solutions. The pH was maintained between 9.5–11 using 10 % NaOH solution, and the temperature was set between 70–95 ºC for 4–5 h under vigorous magnetic agitation. The resulting mixture was then centrifuged for fifteen minutes at 3,000 rpm. The supernatant was then decanted and centrifuged rapidly, until a thick black precipitate was obtained. The precipitate was then washed thoroughly with water and acetone for purification and dried overnight at 100 ºC in hot air oven. The dried samples were then dispersed in double distilled water. The cobalt ferrite NPs were coated with citric acid to impart a negative charge to their surface. The size of CoFe_2_O_4_ NPs was found to be in the range of 10–15 nm by TEM, and the zeta potential value was estimated to be ±34 mV using DLS.

### 4.2. Preparation of Liposome—Nanoparticle Conjugates

Small unilamellar vesicles were prepared by the thin film method. 1 mg of the SOPC lipid was dissolved in 1 ml of chloroform and transferred into a round-bottomed flask. The solvent from the lipid samples was evaporated using a Rotavapor under reduced pressure (1.7 kPa). The dried lipid films were then hydrated with the aqueous iron oxide and cobalt ferrite nanoparticle solutions dispersed in distilled water with the concentration of 1 mg/mL. The final concentration of the lipids was made up to 1 mg/mL. Multilamellar vesicles (MLV) were prepared in our lab by vortexing the lipid suspensions vigorously with glass beads for 10 min. The MLV were further transformed into small unilamellar vesicles (SUV) by sonication for 30 min with 10 s on-off cycles at 50 % amplitude with a Vibracell Ultrasonic Disintegrator VCX 750 (Sonics and Materials, Newtown, CT, USA). To separate the debris from SUV after sonication, the sample was centrifuged for 10 min at 14,000 rpm (Eppendorf Centrifuge 5415C). The control lipid vesicles were prepared in a similar way by dissolving 1 mg of the SOPC lipid in 1 ml of chloroform to form a thin lipid film and the film was hydrated with 1 ml of 20 mM HEPES buffer instead of the nanoparticle solution.

### 4.3. Fluorescence Anisotropy Measurements: Anisotropy and Fluidity

Depending upon the surface charge of the liposomes and the charge of the nanoparticle, the degree of membrane fluidity is altered. The effect of charged nanoparticles in altering the bilayer fluidity of the liposomes can be studied using fluorescent anisotropy probes, such as DPH (diphenyl hexatriene) [50]. The fluorescent dye, DPH, is one of the widely used fluorescent probes to measure the fluidity in native membranes, as well as in artificial membranes and was also used in this work. DPH is a rod-like hydrophobic molecule, which incorporates itself between the fatty acid tails in the core of the lipid bilayer [51]. The optical properties of the DPH largely depends on its environment; it is non-fluorescent in aqueous solutions, whereas after binding to the hydrophobic regions of the bilayer, it shows an intense fluorescence signal [11,52]. A unique feature of the DPH is that its rotational motion and emission intensities are largely dependent on the lipid ordering, and hence, its anisotropy results correlate well with the packing order of the lipids in the bilayer and their fluidity.

In this work, the temperature-dependent fluorescence anisotropy measurements of DPH in control liposomes and liposome-nanoparticle conjugates of zwitterionic SOPC and negatively charged SOPC-POPS liposomes were performed in a 10 mm-path-length cuvette using a Cary Eclipse fluorescence spectrophotometer (Varian, Mulgrave, Australia). The anisotropy values were measured within the temperature range from 15 ºC to 50 ºC by increasing the temperature 5 ºC for every measurement with a time interval of 7 min, with constant mixing at pH 7.0. Varian autopolarizers were used, with slit widths, with a nominal band-pass of 5 nm for both excitation and emission. Ten liters of DPH was added to 2.5 mL of 100 *μ*M solutions of SUV to reach a final concentration of 0.5 *μ*M. DPH fluorescence anisotropy was measured at the excitation wavelength of 358 nm, with the excitation polarizer oriented in the vertical position, while the vertical and horizontal components of the polarized emission light were recorded through a monochromator at 410 nm for both probes. The emission fluorescence of DPH in aqueous solution is negligible. The anisotropy, *<*r*>*, was calculated using the built-in software of the instrument using the below formula:

(13) $$<r>=\frac{I_{||}-I_{⊥}}{I_{||}+2I_{⊥}}$$

where, I*_||_* and I_⊥_ represent the parallel and perpendicular fluorescence emission intensities, respectively.

The values of the G-factor (the ratio of the sensitivities of the detection system for vertically (*I*_HV_) and horizontally polarized light (*I*_HH_)) were determined for each sample separately. The lipid-order parameter, *S*, was calculated from the anisotropy value using the following analytical expression [53]:

(14) $$S=\frac{{\left[1-2\;\left(\frac{r}{r_{0}}\right)+5{\left(\frac{r}{r_{0}}\right)}^{2}\right]}^{\frac{1}{2}}-1+\frac{r}{r_{0}}}{2\;\left(\frac{r}{r_{0}}\right)}$$

where *r*_0_ is the fluorescence anisotropy of DPH in the absence of any rotational motion of the probe. The theoretical value of *r*_0_ of DPH is 0.4, while the experimental values of *r*_0_ lie between 0.362 and 0.394 [53].

### 4.4. Influence of Nanoparticle-Membrane Interactions on Membrane Fluidity

Small unilamellar zwitterionic vesicles were prepared to measure the bilayer fluidity in the presence of positively and negatively charged nanoparticles (NPs). The fluidity of the lipid bilayer membrane of small unilamellar vesicles was determined by measuring the fluorescence anisotropy, which is directly proportional to the lipid ordering in the membrane and inversely proportional to the membrane fluidity. As the membrane becomes more fluid, the mobility of the dye incorporated into the bilayer also increases, whereas the intensity of the fluorescence emission from the dye decreases. Increased anisotropy values indicate that the membrane fluidity is decreased and that the lipids are in a more ordered (liquid) phase. On the other hand, decreased anisotropy values denote increased membrane fluidity, meaning that the bilayer lipids are in a less ordered (liquid) phase (see [54,55]). A decrease in the anisotropy values may also indicate that the dye is incorporated in the liquid state of the bilayer [56].

**Figure 8:**
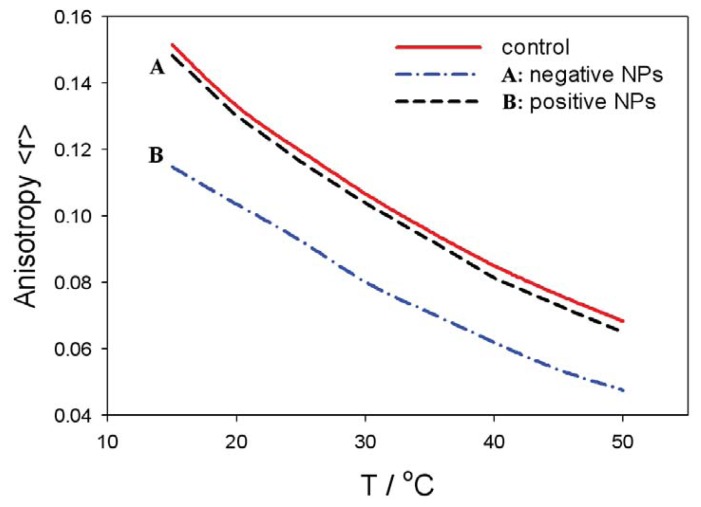
Temperature-dependent fluorescence anisotropy measurement of zwitterionic SOPC bilayer membranes in the presence of negatively or positively charged nanoparticles (NPs). The control curve corresponds to the absence of the nanoparticles.

Figure 8 shows the fluorescence anisotropy measurements of positively and negatively charged NPs in liposomes prepared with the zwitterionic (neutral) lipid (SOPC) surface of liposomes. The anisotropy values are gradually reduced with the temperature in all the cases. The fluidity of zwitterionic SOPC lipid bilayer is increased in the presence of negatively charged NPs, while it is similar to the control in the presence of positively charged nanoparticles (Figure 8). This result coincides well with the theoretically predicted decrease of average lipid dipolar headgroup orientation angle, *< ω >*, in the vicinity of a negatively charged particle (Figure 5, right panel), leading to increased rotational mobility of the lipids, *i.e.*, increased fluidity, as shown in Figure 8. The influence of negatively charged nanoparticles on the average orientation angle, *< ω >*, is possible, due to the attraction between negatively charged nanoparticles and the zwitterionic lipid surface, as presented in Figure 6 (right panel).

On the contrary, the fluidity of the zwitterionic lipid bilayer remains nearly intact in the presence of positively charged nanoparticles (*i.e.*, practically the same as the control values without the nanoparticle), due repulsion between positive nanoparticles and the zwitterionic lipid surface, as shown in the Figure 6 (left panel). The electrostatic repulsion between the zwitterionic surface and positively charged nanoparticles diminishes the probability of the close approach of the nanoparticles to the lipid bilayer surface, and consequently, the average lipid headgroup orientation angle, *< ω >*, is not changed.

## 5. Conclusions

Electrostatic interactions play an important role in determining the efficiency of NP interaction with the lipid bilayer membrane. If attractive electrostatic forces exist between the liposome/cell membrane and the NP surface, a greater amount of NPs will be adsorbed to the membrane, leading to enhanced encapsulation [57,58]. This, in turn, will have a significant effect on the biophysical properties of the membrane. On the other hand, if electrostatic repulsions occur between the liposome/membrane surface and the NPs, a much lesser quantity of NPs will be encapsulated in the liposomes or enter into the cells. When some charged proteins or NPs are adsorbed onto the biological cell surface, the membrane undergoes deformation, and lipids in the constituent bilayers will be reorganized, due to electrostatic interaction between the lipids and NPs/proteins [59–61]. As the cell membrane is negatively charged, positively charged NPs are attracted more towards the surface of the membrane (Figure 3) and show higher levels of internalization when compared to the neutral (zwitterionic) and negatively charged particles [3,62].

The temperature changes induce the phase transition of the membrane lipids [63]. As a result, the NPs, which are incorporated between the fatty acid tails in the membrane, will start to move and vibrate rapidly throughout the bilayer. This dynamic motion of NPs fastens the phase transition of the membrane lipids by reducing the melting temperature and increases the bilayer fluidity. Bothun *et al*. reported that increasing the concentration of silver NPs increases the fluidity of the zwitterionic lipid bilayer membrane. The presence of NPs in the bilayer reduces the pre-transition and melting temperature of the membrane lipids through bilayer disruption [64].

In this paper, we presented within the modified MLPB model an analytical expression for the osmotic pressure between two planar charged surfaces mediated by ions and the ordering of water dipoles. It is shown that in close vicinity to the positively and negatively charged nanoparticles, the average orientation of the lipid headgroups (described by the angle, *< ω >*) is changed, due to electrostatic repulsion/attraction between the positively/negatively nanoparticle and the positively charged trimethylammonium groups of the zwitterionic lipid headgroups, *i.e.*, the lipid headgroups that are in close vicinity to the positively/negatively charged nanoparticle are less/more extended in the direction perpendicular to the membrane surface (Figure 5). It can be further seen in Figures 5 and 6 that the distance between the lipid layer and the nanoparticle (*H*), where the lipid headgroups begin to change their average orientation (Figure 5), coincides well with the distance, *H*, where the osmotic pressure between the lipid monolayer (bilayer) starts to grow/decrease (Figure 6).

The presented theoretical predictions of the influence of charged nanoparticles on the average orientation of the lipid headgroups agree well with the measured fluidity of the zwitterionic lipid bilayer in the presence of positively or negatively charged nanoparticles, as presented in Figure 8. The enhanced interactions between the nanoparticles and zwitterionic liposomes increases the membrane fluidity (Figure 8), which is connected to considerable variation in the lipid ordering [65]. On the other hand, electrostatic repulsions between nanoparticle and the zwitterionic liposome/cell surface leads to much less interaction of the nanoparticles with the membrane lipids, and hence, membrane fluidity is not affected to a considerable extent, as indicated in Figure 8.

## Appendix

### A. Derivation of Osmotic Pressure by Integration of the MLPB Equation

In this Appendix, the expression for osmotic pressure (Equation (4)) between the charged surfaces (as schematically presented in Figure 2) is derived. Using the method of the integration of the Poisson equation to get the osmotic pressure in between the two charged surfaces (see, for example, [32,46,66]), the MLPB Equation (2) will be first integrated, and then, in the second step, the corresponding bulk value of the pressure will be subtracted from the local pressure to get the osmotic pressure, Π. The modified MLPB Equation (2) can be rewritten as (see, also, [30]):

(15) $$-\frac{\text{d}}{\text{d}x}\left[{ɛ}_{0}\;n^{2}\frac{\text{d}\varphi }{\text{d}x}\right]+2\;e_{0}\;n_{0}\;\text{sinh }e_{0}\varphi \beta -n_{0w}\;p_{0}\;\left(\frac{2+n^{2}}{3}\right)\;\frac{\text{d}}{\text{d}x}\left(L(\gamma p_{0}E\beta )\right)=0$$

where Equation (1) was taken into account and *E*(*x*) is the magnitude of electric field strength, as before. Equation (15) is multiplied by *ϕ*′ ≡ d*ϕ/*d*x* and integrated to get the first integral equivalent to the contact theorem:

(16) $$\begin{array}{l} \frac{1}{2}{ɛ}_{0}\;n^{2}E{\left(x\right)}^{2}+2\;n_{0}\;kT\;\left(\text{cosh}(-e_{0}\varphi (x)\beta )\right)-\\ -E(x)\;\left(\frac{2+n^{2}}{3}\right)\;n_{0w}\;p_{0}\;L(\gamma p_{0}E(x)\beta )+\left(\frac{2+n^{2}}{3}\right)\;\frac{n_{0w}}{\gamma \;\beta }\text{ln }\left(\frac{\text{sinh}(\gamma p_{0}E(x)\beta )}{\gamma p_{0}E(x)\beta }\right)=\text{const}. \end{array}$$

where const is osmotic pressure. In the derivation of Equation (16), we used the relations:

$$\int \varphi^{\prime \prime }\;\varphi^{\prime }\;\text{d}x=\int \frac{1}{2}\text{d }{\left(\varphi^{\prime }\right)}^{2}=\frac{1}{2}{\left(\varphi^{\prime }\right)}^{2}\;,\;\int \frac{\text{d}L}{\text{d}x}\varphi^{\prime }\;\text{d}x=L\varphi^{\prime }-\int L\;\text{d}\varphi^{\prime }$$

where *ϕ*″ ≡ d^2^*ϕ/*d*x*^2^ and d*ϕ* = *ϕ*′ d*x*. By subtracting the corresponding bulk values from the local pressure, we obtain the expression for the osmotic pressure difference, Π = *P*_inner_ −*P*_bulk_:

(17) $$\begin{array}{c} \Pi =-\frac{1}{2}{ɛ}_{0}\;n^{2}E{\left(x\right)}^{2}+2\;n_{0}\;kT\;(\text{cosh}(-e_{0}\varphi (x)\beta )-1)-\\ -E(x)\;\left(\frac{2+n^{2}}{3}\right)\;n_{0w}\;p_{0}\;L(\gamma p_{0}E(x)\beta )+\left(\frac{2+n^{2}}{3}\right)\;\frac{n_{0w}}{\gamma \;\beta }\text{ln }\left(\frac{\text{sinh}(\gamma p_{0}E(x)\beta )}{\gamma p_{0}E(x)\beta }\right) \end{array}$$
